# Malignancy in U.S. Air Force fighter pilots and other officers, 1986–2017: A retrospective cohort study

**DOI:** 10.1371/journal.pone.0239437

**Published:** 2020-09-22

**Authors:** Anthony S. Robbins, Sonal R. Pathak, Bryant J. Webber, Roger A. Erich, James D. Escobar, Alisa A. Simon, Shauna L. Stahlman, Kelly J. Gambino-Shirley

**Affiliations:** 1 Public Health and Preventive Medicine Department, U.S. Air Force School of Aerospace Medicine, Wright-Patterson Air Force Base, Ohio, United States of America; 2 DataRev LLC, Atlanta, Georgia, United States of America; 3 Solutions Through Innovative Technologies, Inc., Fairborn, Ohio, United States of America; 4 Aerospace Medicine Department, U.S. Air Force School of Aerospace Medicine, Wright-Patterson Air Force Base, Ohio, United States of America; 5 Armed Forces Health Surveillance Branch, Silver Spring, Maryland, United States of America; Chang Gung Memorial Hospital at Linkou, TAIWAN

## Abstract

**Objective:**

This study sought to determine the incidence rates of cancer, overall and by site, among active component U.S. Air Force fighter pilots, and to compare the rates with those in other active component Air Force officers.

**Methods:**

Using a matched retrospective cohort design, U.S. Air Force fighter pilots were compared with other commissioned officers who entered active component service between 1 January 1986 and 31 December 2006. The cohort was followed for cancer diagnoses in TRICARE and the Veterans Health Administration from 1 October 1995 through 31 December 2017. Fighter pilots and non-fighter pilot officers were compared after matching on sex, age at first observation (15 age groups), and age at last observation (15 age groups). Sex-stratified overall and site-specific cancer rates were compared with matched Poisson regression to determine incidence rate ratios with 95% confidence intervals.

**Results:**

During 1,412,590 person-years of follow-up, among the study population of 88,432 service members (4,949 fighter pilots and 83,483 matched officers), 977 incident cancer cases were diagnosed (86 in fighter pilots and 891 in matched officers). Male fighter pilots and matched officers had similar rates of all malignant cancers (RR = 1.04; 95% CI: 0.83–1.31) and of each cancer site. Female fighter pilots and matched officers also had similar rates of all malignant cancers (RR = 0.99; 95% CI: 0.25–4.04).

**Discussion:**

In the active component U.S. Air Force, fighter pilots and their officer peers had similar overall and site-specific cancer rates.

## Introduction

Military aircrew personnel may experience occupational exposure to galactic cosmic radiation [1, 2], ultraviolet radiation [3], and non-ionizing intra-cockpit radiation [4], leading some to speculate about an increased risk of malignancy [5]. Epidemiologic investigations evaluating the relationship between aviation and cancer in both military and commercial settings have yielded conflicting results: Some studies found no difference in all-site cancer rates between aviators and non-aviators [6–10], one found a higher rate among aviators [5], and one found a lower rate among aviators [11].

In light of these discrepancies, the current study was commissioned to provide an accurate health risk assessment for U.S. Air Force medical policymakers. The objective of this retrospective cohort study was to determine the incidence rate of malignancy—overall and by anatomic site—among active component U.S. Air Force fighter pilots and their officer colleagues, and to establish whether their cancer risks differ.

The U.S. Air Force has approximately 333,000 active duty personnel, of whom nearly 4 percent are pilots [12]. Active duty personnel are entitled to medical care through TRICARE, the insurance program for the U.S. Department of Defense [13]. After being discharged from service, eligible members may also receive care through the U.S. Department of Veterans Affairs [14]. Since 1995, cancers diagnosed in the former have been archived in the Defense Medical Surveillance System, while those diagnosed in the latter have been archived in the Veterans Administration Central Cancer Registry; both systems feature stringent quality-control measures and have been used extensively for epidemiological research [15, 16].

Age-adjusted incidence of some but not all cancers differs between the U.S. military and civilian populations. For example, black servicewomen appear to have lower rates of cervical cancer but higher rates of breast cancer. White servicewomen have lower rates of lung cancer but higher rates of breast cancer. Black and white servicemen have lower rates of lung cancer but higher rates of prostate cancer [17].

## Methods

### Surveillance population

Using Air Force Personnel Center files, an initial cohort of 105,658 officers was constructed of all commissioned officers who entered active component U.S. Air Force service between 1 January 1986 and 31 December 2006 at the rank of second lieutenant, first lieutenant, or captain. The cohort was restricted to commissioned officers to mitigate confounding by socioeconomic status, which has a known association with some cancers [18, 19]. To ensure officers in the 1986 file were newly entering officers, those who were also in the 1985 file were removed from the cohort. Officer occupations were assigned using Duty Air Force Specialty Codes. Since an officer often holds multiple duty codes during a career, the time each officer spent in each duty code was computed, and each officer was assigned to the code in which they spent the most time during their career. Duty Air Force Specialty Codes of 1111, 1115, 11F1, 11F3, and 11F4 identified fighter pilots; the remaining 1,265 codes identified other officers. Some officers (N = 5,987) were classified as non-fighter pilots, although they spent a minority of their Air Force careers as fighter pilots. This corresponds to the “usual occupation” approach used by the National Institute for Occupational Safety and Health for occupational epidemiology. Using this methodology, 4,949 individuals were categorized as fighter pilots and 100,709 as other officers.

### Outcomes

This cohort was followed for incident cancer cases diagnosed from 1 October 1995 through 31 December 2017. Incident cancers were identified in the Defense Medical Surveillance System and the Veterans Affairs Central Cancer Registry. The Defense Medical Surveillance System contains diagnostic codes for all inpatient visits or outpatient encounters occurring at military treatment facilities or at outside facilities reimbursed by TRICARE; these encounters may occur during or after military service. The Veterans Affairs Central Cancer Registry contains cancers diagnosed within Veterans Health Administration facilities. For individuals who entered the cohort between 1 January 1986 and 30 September 1995, all cancers were assumed to be incident cases. For those who entered between 1 October 1995 and 31 December 2017, cancers that were diagnosed prior to cohort entry (n = 12) were considered prevalent cases and excluded.

From TRICARE inpatient and outpatient medical records, potential incident cancer cases were initially identified using *International Classification of Diseases–Ninth Revision* (ICD-9) (before 1 October 2015) or *Tenth Revision* (ICD-10) (beginning 1 October 2015) codes. Potential cases were screened using a cancer case definition algorithm published by the Defense Health Agency [20]. Given the potential of coding errors in the administrative encounter data, all cancer cases were reviewed by physician chart review. Cases that could be definitely confirmed not to be cancer (e.g., miscodes for cancer screening) were recoded. Full chart review methodology has been published elsewhere [21]. All cases from the Veterans Affairs Central Cancer Registry were considered to be confirmed.

Confirmed incident cancers were categorized using rules published by the National Cancer Institute’s Surveillance, Epidemiology, and End Results Program [22]. For analyses of all malignant cancers, only the first cancer case per person was used. For analyses of specific cancer sites, only the first case per site per person was used. Because they are not reportable to central cancer registries, including the Veterans Affairs Central Cancer Registry, basal and squamous cell skin cancers were excluded from the present study.

### Analysis

#### Matching

**Fig 1:**
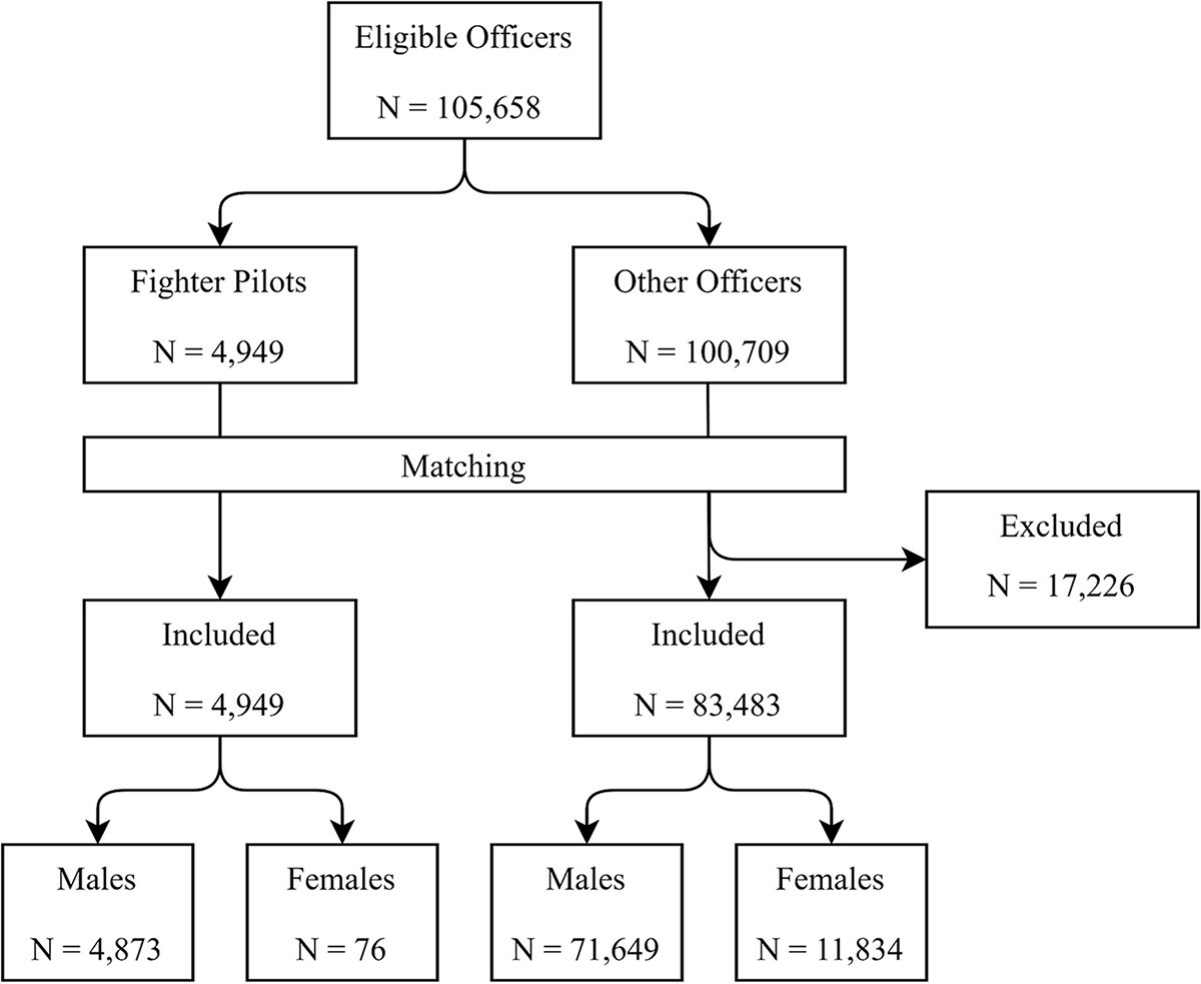
Flow chart of participants, cohort entry years 1986–2006.

Preliminary data analysis revealed that fighter pilots have unique demographic features: They are almost entirely male, usually enter service at younger ages than other officers, and often separate or retire at younger ages. Additionally, upon separation or retirement, they may use employer health insurance rather than TRICARE or the Veterans Health Administration. Consequently, fighter pilots and non-fighter pilot officers, even when stratified by sex, had large and non-overlapping disparities in age at first observation and last observation. Nearly 20% of non-fighter pilot officers had combinations of age at first/last observation that were never observed in fighter pilots (e.g., while 2,373 non-fighter pilot officers entered the cohort at age 30−34 and left the cohort at age 45−49, no fighter pilots had these characteristics). To account for this, a matched cohort design was used, in which fighter pilots and non-fighter pilot officers (hereafter “matched officers”) were compared after matching on sex, age at first observation (15 age groups), and age at last observation (15 age groups). After matching, 4,949 fighter pilots and 83,483 matched officers were available for analysis (Fig 1). Further adjustments were not indicated due to homogeneity of the exposed group. Specifically, all fighter pilots had to meet anthropometric restrictions and medical requirements [23, 24], and 97% self-identified as non-Hispanic white.

#### Calculation of person-time

Person-time at risk was computed differently for individuals who developed and did not develop cancer. For those with cancer, person-time at risk ended on the date of their cancer diagnosis (either first overall cancer or first site-specific cancer, depending on the analysis). For those without cancer, person-time at risk ended on either their last date of military service or the date of their last medical encounter, whichever occurred latest. Person-time at risk was based on all active duty Air Force service, including time in the active component and on activated guard or reserve status.

#### Statistical analysis

We did not sample match officers; for each stratum of fighter pilots, we used all data available from non-fighter pilot officers with the same sex, age at first observation, and age at last observation. Because there was not a fixed ratio of fighter pilots to non-fighter pilot officers across strata, we compared their cancer incidence rates using a generalized estimating equation (GEE) framework within the GENMOD procedure in SAS. These models use a Poisson distributed dependent variable, log link, and log(person-years) offset term. Incidence rate ratios (RRs) with 95% confidence intervals were used to compare the sex-stratified rates of all malignant cancers and specific cancer subtypes among fighter pilots and non-fighter pilot officers. To compare age at first diagnosis of any cancer among fighter pilots and non-fighter pilot officers, we used a GEE model with a normally distributed dependent variable and an identity link. To account for the matched design, a match group variable was included in all models. Baseline characteristics of fighter pilots and non-fighter pilot officers were compared with the chi-square test, with statistical significance set at a two-sided p value of 0.05. All analyses were conducted using SAS version 9.4 (SAS Institute Inc., Cary, NC).

### Regulatory oversight

This study was designated by the Air Force Research Laboratory Institutional Review Board as a “population-based healthcare operations activity for the [U.S. Air Force] medical services” (approval number FWR20180016N). As a non-research activity, in accordance with Department of Defense Manual 6025.18, it was granted waivers of informed consent and Health Insurance Portability and Accountability Act authorization. Identifiable medical records from the Defense Medical Surveillance System and the Veterans Affairs Central Cancer Registry were provided for the outcome ascertainment period of October 1995 through December 2017.

## Results

The study population included 4,949 fighter pilots and 83,483 matched officers followed for a total of 1,412,590 person-years, or a mean of 16.0 years per service member (Table 1). Compared to their matched peers, fighter pilots were more likely to be male (98.5% vs 85.5%; *p*<0.001) and to enter the cohort as a second lieutenant (99.0% vs 85.4%; *p*<0.001).

**Table 1:** Characteristics of U.S. Air Force fighter pilots and matched officers, cohort entry years 1986–2006^a^.

		Fighter Pilots		Matched Officers	
		Male (n = 4,873)	Female (n = 76)	Male (n = 71,649)	Female (n = 11,834)
Rank at entry (%)					
	Second Lieutenant	4,825 (99.0)	75 (98.7)	60,771 (84.8)	10,565 (89.3)
	First Lieutenant	14 (0.3)	1 (1.3)	2,005 (2.8)	458 (3.9)
	Captain	34 (0.7)	0 (0.0)	8,873 (12.4)	811 (6.8)
Total person-years of follow-up		94,742	1,364	1,121,673	194,812
Mean years of follow-up		19.4	18.0	15.7	16.5

^a^Personnel entered the cohort between 1986 and 2006, and were followed for incident cancers from 1995 to 2017.

**Fig 2:**
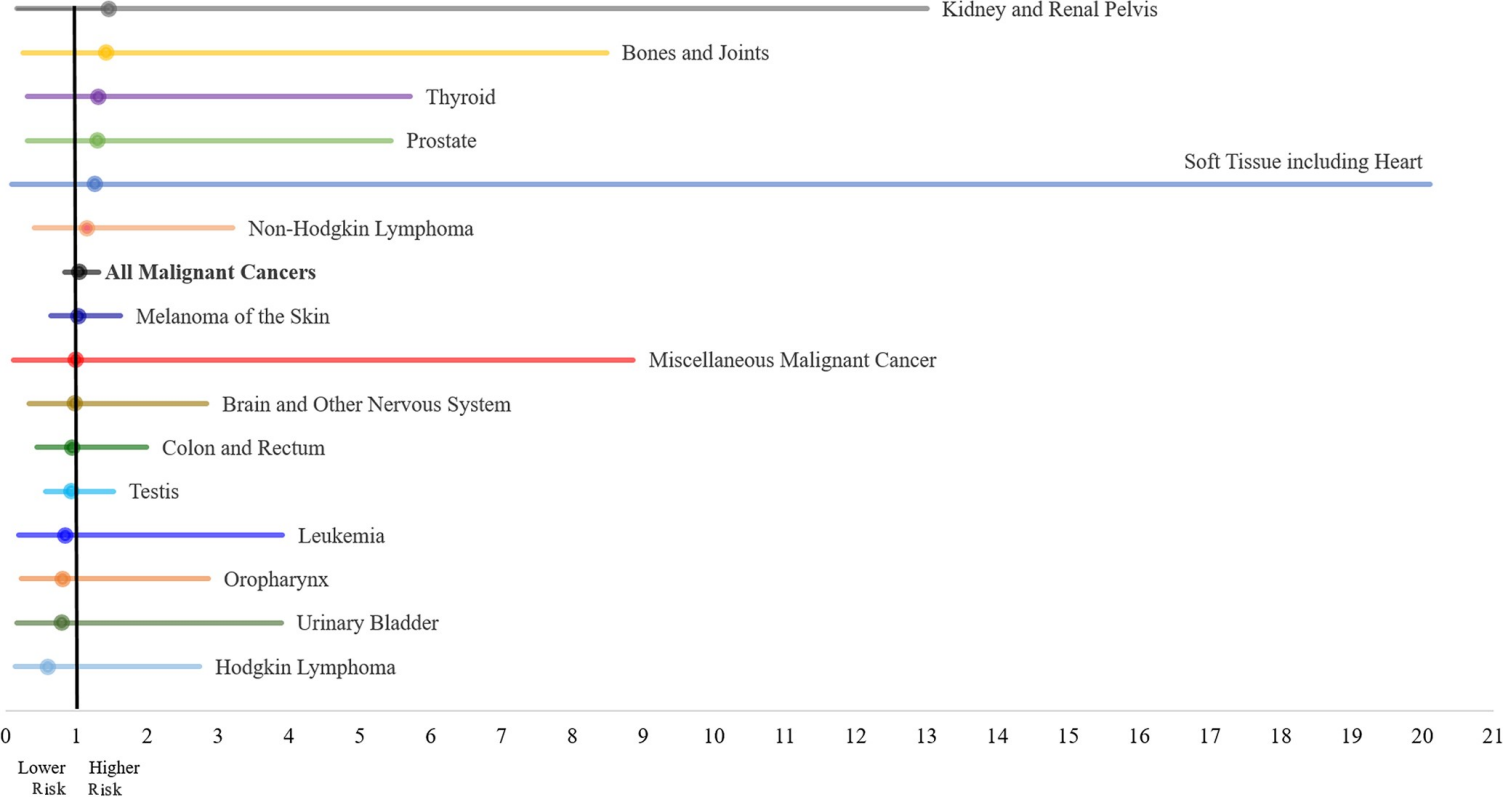
Cancer rate ratios and 95% confidence intervals among male U.S. Air Force fighter pilots and matched officers, diagnosis years 1995–2017.

During follow-up, a total of 977 incident cancers occurred (86 in fighter pilots and 891 in matched officers). Melanoma of the skin, cancer of the testis, cancer of the colon and rectum, and non-Hodgkin lymphoma were the primary cancer types among both male fighter pilots and matched officers; for all other sites, male fighter pilots experienced fewer than 5 cases. Only 2 cancer diagnoses—both melanoma of the skin—occurred in female fighter pilots. Male fighter pilots and matched officers had similar rates of all malignant cancers (RR = 1.04; 95% CI: 0.83–1.31) and of each cancer site. Female fighter pilots and matched officers also had similar rates of all malignant cancers (RR = 0.99; 95% CI: 0.25–4.04) (Table 2; Fig 2). Mean age at diagnosis was 41.6 years for fighter pilots and 41.8 years for matched officers (*p* = 0.73).

**Table 2:** Incident cancer cases among U.S. Air Force fighter pilots and matched officers and rate ratio, diagnosis years 1995–2017^a^.

Sex	Site	Fighter Pilots (n = 85)	Matched Officers (n = 932)	Rate Ratio^b^ (95% CI)
Male	All Malignant Cancers	83	837	1.04 (0.83–1.31)
	Oropharynx	3	15	0.80 (0.22–2.86)
	Colon and Rectum	9	49	0.94 (0.44–1.99)
	Bones and Joints	3	2	1.42 (0.24–8.48)
	Soft Tissue including Heart	1	1	1.26 (0.08–20.1)
	Melanoma of the Skin	24	124	1.02 (0.64–1.62)
	Prostate	2	34	1.29 (0.31–5.44)
	Testis	19	81	0.92 (0.56–1.52)
	Kidney and Renal Pelvis	1	4	1.45 (0.16–13.0)
	Brain and Other Nervous System	4	21	0.97 (0.33–2.84)
	Thyroid	2	16	1.31 (0.30–5.71)
	Urinary Bladder	2	7	0.79 (0.16–3.89)
	Hodgkin Lymphoma	2	12	0.59 (0.13–2.74)
	Non-Hodgkin Lymphoma	5	37	1.15 (0.41–3.21)
	Leukemia	3	10	0.84 (0.18–3.91)
	Miscellaneous Malignant Cancer	1	4	0.99 (0.11–8.86)
Female	All Malignant Cancers	2	95	0.99 (0.25–4.04)
	Melanoma of the Skin	2	5	1.07 (0.20–5.71)

^a^Personnel entered the cohort between 1986 and 2006, and were followed for incident cancers from 1995 to 2017.

^b^Rate ratio comparing fighter pilots to matched officers; each row of the data represents a unique matched group, requiring one cancer case per sex per age at cohort entry per age at cohort exit; consequently, the count of all malignant cancers (for males and females) will exceed the sum of the site-specific cancers.

## Discussion

In a cohort of active component U.S. Air Force officers (n = 88,432) followed for over 1.4 million person-years between 1986 and 2017 (mean of 16.0 years per officer), cancer incidence rates for fighter pilots and matched officers were not significantly different. This finding of no difference extended to all malignant cancers for both male and female officers; in male officers, to the 15 analyzable cancer sites; and, in female officers, to melanoma of the skin. As would be expected in a population of mostly young men, incidence rates were highest for melanoma of the skin and cancers of the testis and colon and rectum—which collectively accounted for 62% of site-specific cancers included after matching.

The null findings presented here are largely in agreement with studies of commercial and military pilots from around the world. Pilots in these studies experienced similar [6–8] or lower [11] overall cancer rates as the populations from which they originated. In some but not all of these studies, rates of individual cancer sites were slightly elevated: bone [6]; prostate [11]; myeloid leukemia [11]; and melanoma of the skin [6, 8]. Small case-control studies of male active duty U.S. Air Force officers found increased odds of brain [25] and testicular cancer [26] among aviators, as compared to non-aviators. The association seen in the former study disappeared after adjustment for socioeconomic status; similar adjustment was not performed in the latter study. A study evaluating prostate cancer incidence in the active duty U.S. Air Force population from 1987 through 2008 found no difference between aviators and non-aviators [27]. A large study of male active duty U.S. Air Force officers from 1975 through 1989 (officers followed an average of 7.1 years), which relied entirely on hospitalization records (apparently not confirmed by chart review), found that aviators had higher rates of all cancers combined, testicular cancer, and bladder cancer, compared to non-flying officers. However, the authors suspected under-adjustment for age differences and recommended further study [5]. In an effort to address these limitations, the present study employed a longer follow-up period, utilized data from medical encounters during and after active duty military service, confirmed diagnoses via chart review, incorporated cases captured in the central cancer registry of the Veterans Health Administration, and controlled for important confounding variables (i.e., sex and age at entry and exit from the cohort) by applying a matched study design and analysis.

This study has a number of limitations. First, capture of cancer cases may have been incomplete. Individuals with cancer who did not seek care, or who sought care outside TRICARE or the Veterans Health Administration, would not be discovered in the available datasets. It is not readily apparent if outcome misclassification would differ between fighter pilots and matched officers. The Automated Central Tumor Registry, the central cancer registry of the Department of Defense, was not utilized due to under-capture of incident cancer cases during the surveillance period [17, 28]. Second, Air Force Personnel Center data files have known demographic inconsistencies, with service members reporting multiple sexes, races, and dates of birth through the course of their careers. Using previously developed standardized processes [29], the last record per person was used to obtain sex, race, and date of birth. Third, while we matched on age at first and last observation, we could not account for the true difference in occupational exposure between the two groups. Duration of time as a fighter pilot—and, more specifically, in a fighter cockpit—would be a better measure of exposure, but data quality issues prevented the use of flight hours in this analysis. Since the mean age at diagnosis was nearly identical between fighter pilots (41.6 years) and their non-fighter pilot peers (41.8 years), it seems unlikely that stratifying the former group by flight experience would significantly alter the results.

Although this study found no difference in cancer rates between U.S. Air Force fighter pilots and matched officers who entered active component service between 1986 and 2006, it does not minimize the significance of cancer diagnoses in any particular group. A single diagnosis of cancer can reverberate throughout a tight-knit community, such as military fighter pilots, and several diagnoses within the community may appear to constitute an epidemic. These anecdotal experiences may catalyze important epidemiologic research. The present study attempts to address this issue objectively, but its findings should be interpreted in light of the above limitations. Given the prolonged latency period of many cancers, future studies should track individuals for longer periods after separation from military service. These studies should maximize case ascertainment and account for socioeconomic differences and other confounding variables between fighter pilots and control populations.
